# Assessing outcomes for cost-utility analysis in mental health interventions: mapping mental health specific outcome measure GHQ-12 onto EQ-5D-3L

**DOI:** 10.1186/s12955-016-0535-2

**Published:** 2016-09-20

**Authors:** Marie Lindkvist, Inna Feldman

**Affiliations:** 1 Department of Public Health and Clinical Medicine, Epidemiology and Global Health, Umeå University, Umeå, Sweden; 2 Department of Statistics, Umeå University, Umeå, Sweden; 3 Department of Women’s and Children’s Health, Uppsala University, Uppsala, Sweden

**Keywords:** Mapping, Preference-based measures, Mental health, Quality of life, Utilities

## Abstract

**Background:**

Many intervention-based studies aiming to improve mental health do not include a multi-attribute utility instrument (MAUI) that produces quality-adjusted life-years (QALYs) and it limits the applicability of the health economic analyses. This study aims to develop ‘crosswalk’ transformation algorithm between a measure for psychological distress General Health Questionnaire (GHQ-12) and MAUI EuroQoL (EQ-5D-3L).

**Methods:**

The study is based on a survey questionnaire sent to a random sample in four counties in Sweden in 2012. The survey included GHQ-12 and EQ-5D instruments, as well as a question about self-rated health. The EQ-5D index was calculated using the UK and the Swedish tariff values. Two OLS models were used to estimate the EQ-5D health state values using the GHQ-12 as exposure, based on the respondents (*n* = 17, 101) of two counties. The algorithms were applied to the data from two other counties, (*n* = 15, 447) to check the predictive capacity of the models.

**Results:**

The final models included gender, age, self-rated health and GHQ-12 scores as a quantitative variable. The regression equations explained 40 % (UK tariff) and 46 % (Swedish tariff) of the variances. The model showed a satisfying predictive capacity between the observed and the predicted EQ-5D index score, with Pearson correlation = 0.65 and 0.69 for the UK and Swedish models, respectively.

**Conclusion:**

The algorithms developed in this study can be used to determine cost-effectiveness of services or interventions that use GHQ-12 as a primary outcome where the utility measures are not collected.

## Background

Well-being is an important determinant of health and social outcomes. Health influences wellbeing and wellbeing itself influences health, thus health is one of the top things people say matters for wellbeing [1]. According to [2], both physical health and mental health can influence wellbeing. Mental health is defined as a state of well-being while the positive dimension of mental health is stressed in WHO’s definition of health: “Health is a state of complete physical, mental and social well-being and not merely the absence of disease or infirmity” [3]. Nevertheless, mental health problems account for a substantial burden of disease globally, with the World Health Organization predicting that by 2030, mental health problems will be the highest-ranking disease in terms of burden in affluent countries [4]. Consequently, numerous researchers have designed and evaluated diverse interventions that aim to improve mental health among different population groups. We can name risk management interventions [5, 6], community programs to improve health behaviors and mental well-being [7], parenting programs [8, 9], healthcare strategies [10, 11] and treatments [12], and many others. These interventions are carried out in different societal sectors (government, municipality, healthcare, etc.); however, the same instrument, General Health Questionnaire (GHQ-12), is used when evaluating their efficacy/effectiveness. The General Health Questionnaire (GHQ) is a measure of the current mental health, and since its development by Goldberg in the 1970s [13], it has been extensively used in different settings and different cultures. GHQ-12 is often used according to a common approach to assess the outcomes of health interventions, that is, to obtain personally reported description of mental health status across various dimensions and then to apply a numerical scoring system.

At the same time, the implementation of effective interventions is often questioned because of the scarcity of available resources to meet the growing demands for healthcare and social services. That is why the interest in economic evaluation as a tool to inform resource allocation has increased over time. The aim of economic evaluation of different interventions targeted mental health problems is to allow the comparison of the cost-effectiveness of services in different societal sectors, such as healthcare, municipality care, public health interventions, etc. In this way, decision makers can be informed about where the greatest net benefits could be obtained. The technique of cost-utility analysis allows for such comparisons, both within and across societal sectors. In this framework, costs are measured in monetary terms and outcomes are measured in a generic common unit such as quality-adjusted life-years (QALYs). There are a number of multi-attribute utility instruments (MAUIs) that measure health-related quality of life, but they uniquely have a ‘utility’ algorithm that converts people’s responses to a single utility score measured on a 0–1 scale, where 0 denotes death and 1 denotes the best health outcome measured by the instrument. The utility scores produced by these instruments, in principle, measure the strength of the people’s preference for the health state. To obtain QALYs, the utility of a health state is multiplied by the length of time spent in the particular health state. The most commonly used MAUI for evaluating both the mental and physical disorders is the EuroQoL–five dimension (EQ-5D). Its advantages are both brevity and simplicity, comparison with other commonly used MAUI like SF-6D, HUI, AoQL-8D, and others [14]. Nonetheless, GHQ-12, which is often used in evaluating mental health promotion interventions, cannot be used in cost-utility analyses to estimate cost per quality adjusted life year (QALY), as it is not preference-based. As was mentioned by Brazier et al. (2010), “this lack of use of generic preference-based measures is a barrier to population economic models with the best evidence on effectiveness” [15]. The authors suggest using the mapping technique as a possible solution to the predicted health state utility values when only a no preference-based measure, like GHQ-12, has been included in the study. This approach requires that the two measures be administered on the same population, and it involves estimating the relationship between a non-preference-based measure and a generic preference-based measure using so called ‘cross-walking’ [16]. Typically, mapping uses two datasets: an estimation dataset that contains respondents’ self-reported scores for their own health using two or more preference and non-preference-based measures, and a study dataset containing only the non-preference-based measure. Regression techniques are usually used on the estimation dataset to determine a statistical relationship between the measures, and the results are then applied to the study dataset to obtain predicted health state utility values.

To the best of our knowledge, there are three studies estimating mapping functions from specific mental health measures into generic preference-based measures of health. The study by Brazier et al. [17] presented mapping functions between GHQ-12 and SF-6D using the total GHQ-12 score and the items to predict the SF-6D scores. Analyses were based on groups of people with mental health problems, from moderate to severe. Another study by Mihalopoulos et al. [18] compared the sensitivity of five commonly used MAUIs (including EQ-5D) with disease-specific depression outcome measures and developed ‘crosswalk’ transformation algorithms between the measures. Both studies aimed to estimate the functions to predict the MAUIs scores from mental health-specific measures commonly used in the mental health services. The third study by Serrano-Aguilar et al. [19] is only one study that estimates the relationship among mental health status measured by GHQ12, Health Related Quality of Life (EQ-5D), and Health-State Utilities in a general population [19]; however, the findings have limited applicability because the authors did not follow the recommendations [15]; specifically, they did not include other health measures in the model, and the model was not applied to another population to test the predictability and accuracy.

The aim of this study is to assess the relationship between the commonly used measures for psychological distress General Health Questionnaire (GHQ12) and MAUI EuroQoL (EQ-5D-3L), and develop ‘crosswalk’ transformation algorithm between the measures. This algorithm can be used to determine the cost-effectiveness of services or interventions that use GHQ-12 as a primary outcome, where the utility measures are not collected.

## Method

### Instrument description

#### EQ-5D-3L

The 3L version of the EQ-5D questionnaire is the standard version that has been used in hundreds of clinical trials and methodological studies published in the peer-reviewed literature [20]. It is a brief self-reported measure of generic health that consists of five dimensions (mobility, self-care, usual activities, pain/discomfort, and anxiety/depression), each with three levels of functioning (e.g., no problems, some problems, and extreme problems). This health state classifier can describe 243 unique health states that are often reported as vectors ranging from 11,111 (full health) to 33,333 (worst health). Numerous societal value sets have been derived from the population-based valuation studies around the world which, when applied to the health state vector, result in a preference-based score that typically ranges from states worse than dead (>0) to 1 (full health), anchoring dead at 0.

#### EQ-5D-3L value set

Research carried out by EuroQol Group members has concentrated on statistical modeling to generate values for all the 243 theoretically possible health states defined by EQ-5D-3L. A set of weights that represent the general population’s preferences might be the ideal system of choice, but such a system is not always available. A country-specific value set for EQ-5D health states was first generated in the UK [21] based on hypothetical values derived from a sample of the general population. The National Institute for Health and Clinical Excellence (NICE) in England and Wales recommends using this UK EQ-5D ‘social tariff’ for QALY weightings [22]. In the absence of a set of national population-based utility weights, the majority of cost-effectiveness research adopted the UK value set.

In Sweden, the Dental and Pharmaceutical Benefits Agency (TLV) states that QALY weightings can be based either on direct or indirect measurements (‘where a health classification system such as EQ-5D is linked to QALY weightings’) and that ‘QALY weightings based on appraisals of persons in the health condition in question are preferred before weightings calculated from an average of a population estimating a condition depicted for it (e.g., the ‘social tariff’ from EQ- 5D)’ [23]. It means that TLV prefers experience-based rather than hypothetical values. A recently published study by Burström et al. [24] presented the estimations of experience-based Swedish value set for EQ-5D-3L health states.

In our study, we use two different value sets for EQ-5D health states:

1. The UK value sets, based on the *hypothetical* values derived from a sample of the general population [21] and
2. The Swedish *experience-based* value sets for EQ-5D health states derived from a general population health survey data [24].

#### GHQ-12

GHQ-12 is one of the most widely used screening tests to detect psychiatric morbidity in community settings and non-psychotic psychiatric disorders in clinical settings, and it is designed as a structured, brief, and self-administered questionnaire [13]. Every one of its 12 items regarding recent symptoms, feelings, or behaviors is answered on a four-category Likert scale. Categories 1 and 2 are given value 0, and categories 3 and 4 are given value 1. Values from 12 items are added together to get an overall score. A probable psychiatric case is considered when the score is equal to or greater than 3.

#### The SRH question

Self-rated health (SRH) was measured by the question: “How do you rate your general health?” with the options ‘very good,’ ‘good,’ ‘neither good nor poor,’ ‘poor,’ and ‘very poor.’

### Material/study population

Data were obtained from the cross-sectional postal survey questionnaires, conducted during March–May 2012. The surveys were addressed to random population samples of men and women, aged 16–84 years, from 39 municipalities in 4 counties in the central part of Sweden. Together, the four counties have about one million inhabitants in this age range. The sampling was random and stratified by gender, age group, and municipality; the response rate was 51 %. The data collection was completed after two postal reminders. Corresponding surveys have been undertaken in 2000, 2004, and 2008 [25, 26]. The respondents gave their informed consent so that questionnaire data could be linked to the Swedish official registries through the individuals’ personal identification numbers. All handling of personal identification numbers was carried out by Statistics Sweden, the statistical administrative authority in Sweden.

**Fig. 1:**
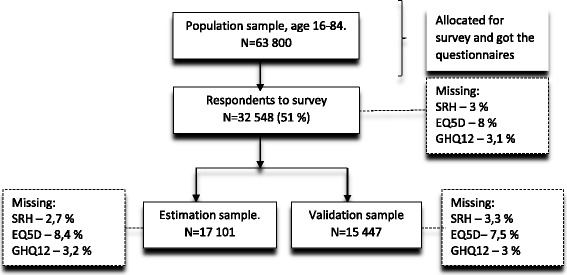
Population survey samplings results used in the study

The EQ-5D-3L self-report descriptive system was transformed into utility values using the English (EQ-5D-UK) and Swedish (EQ-5D-SW) value sets. The General Health Questionnaire (GHQ-12) and a self-rated health (SRH) questionnaire were included in this study, along with information about age and sex. The total study sample included 32,548 respondents, while data from respondents of two counties (Estimation sample, *n* = 17,101) were used for the statistical analyses and deriving of cross-work algorithms. The algorithms were applied to the survey data of the respondents from another two counties, (Validation sample, *n* = 15,447) to check the predictive capacity of the models. The survey sampling results are presented on Fig. 1.

**Fig. 2:**
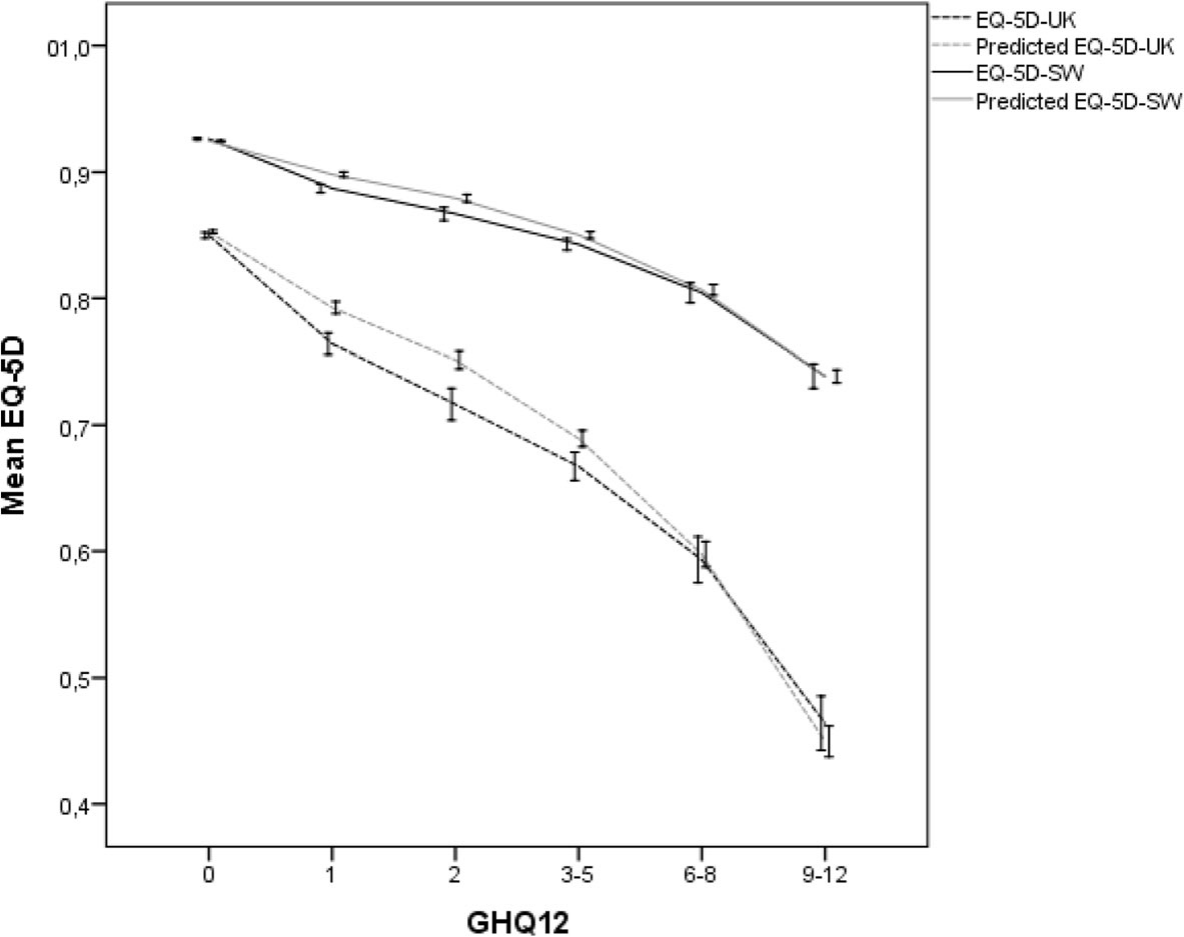
Observed and Predicted values of EQ-5D and EQ-5D-SW with 95 % error bars against GHQ-12 (Validation sample)

### Analyses

All analyses were performed with IBM SPSS Statistics, version 23.

#### Total sample

For description of the scales: mean, standard deviation, median, range, ceiling effect, floor effect, and skewness were used. The distribution of the categorical variables was described with numbers and percentages. For the description of the relation between the GHQ-12 and health utility, the mean and standard deviation was calculated for each level of GHQ-12. Description of variables in the two samples were performed with number and percentages for categorical variables and with mean and standard deviation for quantitative variables. Test of difference between the two subsamples were performed with Pearson Chi-square test for categorical variables and with independent samples *t*-test for quantitative variables. Further, Cohen’s effect size measure was calculated for quantifying the difference between the subsamples regarding the scale means.

#### Estimation sample

The aim was to build two models, one that related the GHQ-12 to the EQ-5D-UK and one that related the GHQ-12 to the EQ-5D-SW. Health utility measures often show a truncation effect where a proportion of the individuals achieve the upper bond. However, when the intention of the model is economic evaluation, OLS used with robust standard errors is recommended as a simple and valid approach [27]. Hence, the Huber sandwich estimator was used to estimate the standard errors, which gives robust estimates even if the underlying model is incorrect. OLS-models were used to develop the transformations between the health utility (EQ-5D-UK and EQ-5D-SW) and the explanatory variables GHQ12 (as a quantitative variable), self-rated health, age, and sex. The interaction between the explanatory variables regarding the effect on health utility was investigated by including interaction terms in the models. Evaluation of goodness-of-fit for different models was performed with R^2^ and RMSE (root mean squared error). The final models were presented with parameter estimates, and robust standard errors calculated with the Huber sandwich estimator.

#### Validation sample

Capacity checking of the models was performed by calculating Pearson correlation between the observed and predicted values of health utility. Further evaluations of the models were done by computing the mean and 95 % confidence interval of the observed and the predicted values of the health utility measures and for the absolute errors. Forecast errors were computed by dividing the absolute error by the mean of the observed health utility [28]. The forecast errors were presented by the mean and 95 % confidence intervals. Finally, the observed and predicted values for the EQ-5D-UK and EQ-5D-SW were plotted against the GHQ-12, divided into categories.

#### Estimation sample and validation sample

A sensitivity analysis of the results was performed by analyzing the pattern of missing values in the data and performing an iterative Markov chain Monte Carlo (MCMC) for imputing missing values. Then the model specification and capacity checking was performed again for data with imputed values and those results was compared to the original results.

## Results

### Total sample

Descriptive information regarding the distributions of the EQ-5D-UK, EQ-5D-SW, and GHQ-12 are presented in Table 1.

**Table 1:** Description of EQ-5D-UK, EQ-5D-SW and GHQ-12 (Total sample, *n* = 32,548)

EQ-5D-UK: Possible values: −0.59 (poorest health) to 1 (best health)	
Missing: 8.0 % (*n* = 2602)	
Mean (SD)	0.80 (0.22)
Median (range)	0.80 (−0.59 to 1)
% Floor (−0.59)	0.03 % (*n* = 10)
% Ceiling (1)	33.4 % (*n* = 10,003)
EQ-5D-SW: Possible values: 0.34 (poorest health) to 0.97 (best health)	
Missing: 8.0 % (*n* = 2602)	
Mean (SD)	0.90 (0.09)
Median (range)	0.93 (0.34–0.97)
% Floor	0.03 % (*n* = 11)
% Ceiling	33.4 % (*n* = 10,002)
GHQ-12: Possible values: 12 (poorest health) to 0 (best health)	
Missing: 3.1 % (*n* = 1008)	
Mean (SD)	1.10 (2.42)
Median (range)	0 (0 to 12)
% Floor (12)	0.9 % (*n* = 290)
% Ceiling (0)	71.2 % (*n* = 22,448)

The health utility transformed with Swedish weights (EQ-5D-SW) gives higher values with smaller variability compared to health utility transformed with English weights (EQ-5D-UK). Both the EQ-5D measures showed an apparent truncation effect, with a ceiling effect of 33.4 % (highest value 1 for EQ-5D-UK and 0.97 for EQ-5D-SW). GHQ-12 had a ceiling effect of 71.2 %, which is very high. The relation between the GHQ-12 and the outcomes EQ-5D-UK and EQ-5D-SW for the total sample is shown in Table 2.

**Table 2:** Relation between GHQ12 and the outcomes EQ-5D-UK and EQ-5D-SW (Total sample, *n* = 32548^a^)

	GHQ-12	EQ-5D-UK	EQ-5D-SW
	n (%)	Mean (SD)	Mean (SD)
GHQ-12			
0	20,836 (71.4)	0.85 (0.17)	0.93 (0.06)
1	2626 (9.0)	0.76 (0.22)	0.89 (0.09)
2	1458 (5.0)	0.71 (0.25)	0.87 (0.10)
3	910 (3.1)	0.69 (0.25)	0.85 (0.10)
4	724 (2.5)	0.67 (0.26)	0.84 (0.11)
5	553 (1.9)	0.63 (0.28)	0.83 (0.12)
6	463 (1.6)	0.63 (0.29)	0.82 (0.12)
7	353 (1.2)	0.58 (0.31)	0.80 (0.14)
8	286 (1.0)	0.54 (0.32)	0.78 (0.14)
9	239 (0.8)	0.53 (0.33)	0.77 (0.14)
10	223 (0.8)	0.54 (0.32)	0.77 (0.14)
11	246 (0.8)	0.44 (0.31)	0.73 (0.14)
12	270 (0.9)	0.36 (0.35)	0.69 (0.16)

^a^GHQ-12 3.1 % missing, EQ-5D-UK 8.0 % missing, EQ-5D-SW 8.0 % missing

The EQ-5D measures have higher values (indicated better health) for low values on the GHQ-12 and declines in a stepwise manner when the GHQ-12 values become higher. Table 3 provides the background characteristics of the two subsamples (Subsample1: Model building, Subsample 2: Capacity checking). The distributions for sex and self-rated health and the mean values for age were almost identical in the two subsamples. The mean values for EQ-5D-UK and EQ-5D-SW are similar with p-values for differences 0.985 and 0.184 respectively. The mean value for GHQ-12 is somewhat higher in Subsample 1 with a *p*-value of 0.001. However, Cohen’s effect size measure is 0.04 which is considered trivial.

**Table 3:** Characteristics of the individuals (Total sample, *n* = 32548^a^)

	Estimation sample	Validation sample	*p*-value^b^	Effect size^c^
	Model building *n* = 17,101	Capacity checking *n* = 15,447		
Sex, n (%)				
Men	7874 (46.0)	7009 (45.4)	0.226	
Women	9227 (54.0)	8438 (54.6)		
Self- rated health, n (%)				
Very good	3048 (18.3)	2693 18.0)	0.932	
Good	8191 (49.2)	7401 (49.6)		
Neither nor	4354 (26.2)	3901 (26.1)		
Bad	893 (5.4)	782 (5.2)		
Very bad	159 (1.0)	147 (1.0)		
Age, Mean (SD)	55.44 (19.44)	55.48 (18.52)	0.871	
EQ-5D-UK, Mean (SD)	0.80 (0.22)	0.80 (0.22)	0.985	0.00
EQ-5D-SW, Mean (SD)	0.90 (0.09)	0.90 (0.09)	0.184	−0.01
GHQ-12, Mean (SD)	1.14 (2.46)	1.05 (2.38)	0.001	0.04

^a^Self-rated health 3.0 % missing, EQ-5D-UK 8.0 % missing, EQ-5D-SW 8.0 % missing, GHQ-12 3.1 % missing

^b^Pearson Chi-square test for categorical variables and independent samples *t*-test for quantitative variables

^c^Cohen’s effect size measure

### Model building (estimation sample)

To develop crosswalk transformation algorithms from the GHQ-12 to the health utility measures, the two EQ-5D measures were regressed with OLS upon GHQ-12, self-reported health, age, and sex in stepwise procedure. Evaluations of different models are presented in Table 4 with goodness of fit measures. First, the binary models for the GHQ-12 and self-reported health were performed. Then, the algorithms with all four explanatory variables were performed, also with interaction terms.

**Table 4:** Model construction (Estimation sample)

	R^b^	RMSE^a^
EQ-5D-UK^b^		
GHQ	0.181	0.200
SRH	0.407	0.170
GHQ + SRH + Age + Sex	0.449	0.164
EQ-5D-SW^b^		
GHQ	0.241	0.077
SRH	0.437	0.071
GHQ + SRH + Age + Sex	0.503	0.063

^a^Lower values indicates better model fit

^**b**^No important interaction effect was detected between the variables

The analyses showed that no important interaction effects between the explanatory variables in relation to the health utility measures were present. Inclusion of self-reported health together with GHQ-12 was essential for acceptable values on R^2^ and the following algorithms were chosen for the crosswalk transformations (self-rated health = SRH; overall score of GHQ-12 = GHQ):

#### Model EQ-5D-UK

0.987 - 0.001*Age + 0.025*(Gender = Man) - 0.074*(SRH = Good) - 0.200*(SRH = Neither nor) -0.444*(SRH = Bad) - 0.660*(SRH = Very bad) - 0.019*GHQ.

#### Model EQ-5D-SW

0.972 - 0.0004*Age + 0.010*(Gender = Man) - 0.020*(SRH = Good) - 0.076*(SRH = Neither nor) -0.182*(SRH = Bad) - 0.261*(SRH = Very bad) - 0.010*GHQ.

Full descriptions of the models are presented in Table 5.

**Table 5:** Models (Estimation sample)

	EQ-5D-UK^*^		EQ-5D-SW^*^	
Variable	Coef	Std. Error	Coef	Std. Error
Intercept	0.987	0.0045	0.972	0.0017
Age	−0.001	<0.0001	−0.0004	<0.0001
Gender				
Woman	*ref*		*ref*	
Man	0.025	0.0027	0.010	0.0011
Self-rated health				
Very good	*ref*		*ref*	
Good	−0.074	0.0026	- 0.020	0.0008
Neither nor	−0.200	0.0041	- 0.076	0.0016
Bad	−0.444	0.0117	- 0.182	0.0045
Very bad	−0.660	0.0282	−0.261	0.0114
GHQ	−0.019	0.0009	−0.010	0.0004

^*^All estimates had a *p*-value < 0.001

### Capacity checking (validation sample)

Validation of the models are presented in Table 6.

**Table 6:** Capacity checking of the models (Validation sample)

	EQ-5D-UK		EQ-5D-SW	
Pearson correlation^a^	0.678 (*p* < 0.000)		0.715 (*p* < 0.000)	
	Mean (CI)		Mean (CI)	
Observed values	0.800 (0.796, 0.803)		0.904 (0.902, 0.905)	
Predicted values	0.808 (0.806, 0.810)		0.904 (0.903, 0.905)	
Absolute error	0.115 (0.113, 0.117)		0.042 (0.041, 0.043)	
Relative forecast error	14.4 % (14.1, 14.6)		4.6 % (4.5, 4.7)	
Observed values	≤0.8	>0.8	≤0.8	>0.8
Relative forecast error	15.5 % (15.1, 15.8)	12.8 % (12.5, 13.0)	13.6 % (13.2, 14.0)	3.6 % (3.5, 3.6)

^a^Pearson correlation between observed and predicted values of health utility

Pearson correlation between the observed and predicted values for the EQ-5D-UK and EQ-5D-SW were 0.678 and 0.715, respectively. The mean absolute error and the mean relative forecast error were smaller for the transformation with the Swedish weights (MAE = 0.115 for EQ-5D-UK and MAE = 0.042 for EQ-5D-SW). The relative forecast error was larger for the smaller observed values on the EQ-5D measure and also smaller for the transformation with the Swedish weights. Overall, the results from the validation of the models (Table 6) are in line with or better than previous research [17, 19, 28]. Figure 2 provides the observed and predicted values for the EQ-5D-UK and EQ-5D-SW plotted against the GHQ in six categories. The predictions are best for the low (GHQ = 0) and the upper half (GHQ = 6 to GHQ = 12) and somewhat poorer in between.

### Sensitivity analysis (estimation sample and validation sample)

An analysis of the pattern of missing values in the data revealed that the pattern was arbitrarily and an iterative Markov chain Monte Carlo (MCMC) method was used for imputing missing values. Regression analyses with EQ-5D-UK and EQ-5D-SW as outcomes were performed for the data with imputed values, and capacity checking for those models was performed by calculating relative forecast errors (Table 7). A comparative analysis between data with and without imputed values showed similar results.

**Table 7:** Sensitivity analysis with imputed values for GHQ-12, EQ-5D-UK, EQ-5-SD and Self-rated health (Validation sample)

	EQ-5D-UK		EQ-5D-SW	
Relative forecast error	14.7 % (14.4, 14.9)		4.7 % (4.6, 4.8)	
Observed values	≤0.8	>0.8	≤0.8	>0.8
Relative forecast error	15.8 % (15.5, 16.2)	13.0 % (12.8, 13.2)	13.1 % (12.7, 13.5)	3.7 % (3.6, 3.7)

## Discussion

### Main findings

In this study we have developed transformation algorithms between the non-preference-based mental health specific outcome measure GHQ-12 and the generic health utility instrument EQ-5D-3L. These mapping algorithms provide a practical solution for researchers seeking to use existing data sets with GHQ-12 data, but where no preference-based utility measure is used, to facilitate an economic evaluation. Three additional variables were included in the final algorithms: age, gender, and self-rated health to increase the performance of the model. We do not think that this is a limitation because such data are usually collected during the evaluation of the mental health interventions. Different models were constructed 1) for the UK value sets, based on the *hypothetical* values and 2) for the Swedish *preference based* value set, to increase the applicability and practical use of the study. The prediction capacity of the Swedish values based model was slightly better than the UK value based one, but both models have shown the same pattern in the error degree with the good predictive results observed for the low and the upper half of the GHQ-12 score and poorer in between. It means that the accuracy of the deriving quality of life utilities is better for severe mental health problems (in our case, when GHQ-12 scores are higher than 3). These results, however, are in contrast to previous observations that the degree of error tends to be larger when the health condition gets more severe, and the utilities are usually overestimated. In agreement with previous studies [15], we found a simple additive model with the utility score as the dependent variable and the GHQ-12 scores as independent variables to be the most appropriate functional form, with the additional patient characteristics such as age, gender, and self-rated health having a positive impact on the model’s performance.

Despite concerns over the use of OLS, we find this method of estimation to be suitable in this case. A simulation study [27] showed that when the intention is to provide an economic evaluation and the true utilities are bounded at 1, then the OLS model coupled with robust standard errors is a simple and valid approach. A recent review of crosswalk studies between MAUIs and other measures [15] found that the explanatory power of studies ranged from an R2 of 0.17 to 0.71, with the majority between 0.4 and 0.5. For example, Mihalopoulos et al. [18] reported correlation coefficients between depression-specific outcome measures and MAUI EQ-5D-5L between 0.45 and 0.69. By these standards, the crosswalk between the mental health specific outcome measure GHQ-12 and MAUI EQ-5D-3L in this study performs well.

### Strength and limitations

The study is based on the large community based samples aimed at giving representative pictures of health conditions in a general Swedish population with strong statistical power. Two independent subsamples with the same population profiles were used, one to construct the model and another to check the models capacity. This technique strengths the credibility and robustness of the developed algorithms. However, the response rates of 51 % pose a risk of bias in the results, as non-participation in health surveys has been shown to be associated with poor health [29]. Nonetheless, there were fewer participants in the younger age group (16–24) compared with the general population. The survey was also a cross-sectional design, thus, a comparison of the responsiveness of the instruments to change over time could not be assessed.

The proposed model cannot be applied using only the data set with complete responses to the GHQ-12, which requires additional data on age, gender, and self-rated health. It was shown that the overlap between the GHQ12 - items and the EQ-5D-3L is very limited, since they only share the anxiety/depression dimension [30]. That is why including of the self-rated health variable into the model is absolutely necessary, to take into account four other “physical” dimensions of the EQ-5D. That means that the pertinence of the algorithms is limited to the studies included self-rated health questionnaires additionally to the GHQ-12 survey.

Finally, although this study presents a technique for deriving utility from the GHQ-12 instrument, while MAUI is not included, it is important to appreciate that this is a second best solution to the inclusion of such an instrument in a study that aims to conduct a health economic evaluation. Predicted utilities cannot create or estimate the content that is not in the mental health specific instrument, rather they can only transform the content of the instrument to a second best estimate. Even though the current study has provided an internal validity of the mapping algorithms, external validation is still required, although the big sample size and the international context of the study help to reduce the risks in external validity.

## Conclusion

Our paper presents a set of algorithms to map from the mental health specific instrument GHQ-12 to the health state values, which will facilitate the cost-effectiveness studies in this area. The models are reasonably simple, in that any data set with complete responses to the GHQ-12 together with the respondent’s age, gender, and self-rated health can be used to predict the EQ-5D. The models presented in this paper can be used to estimate the mean EQ-5D-3L values in other samples. However, future work is required to assess whether our models would perform as well in some special pollution groups.

## Abbreviations

EQ-5D-3L: EuroQoL–five dimensions three levels multi-attribute utility instrument
GHQ-12: General health questionnaire 12 items
QALY: Quality-adjusted life-years
